# Severe synergistic toxicity from docetaxel in a patient treated concurrently with protease inhibitors as part of HIV post-exposure prophylaxis: a case report

**DOI:** 10.4076/1752-1947-3-8866

**Published:** 2009-08-27

**Authors:** Madeleine Hewish, Rowan Miller, Martin Forster, Ian Smith

**Affiliations:** 1 Department of Medicine, Royal Marsden Hospital, Fulham Road, London, SW3 6JJ, UK

## Abstract

**Introduction:**

Docetaxel is a semisynthetic taxane commonly used in solid tumour oncology. Its pharmacokinetics has been widely studied, and it is well established that it is metabolized to pharmacologically inactive products by the cytochrome P450 3A iso-enzymes. However, there have been few reports of the consequences of drug interactions between taxanes and other drugs metabolized by the cytochrome P450 pathway. To the best of our knowledge, this is the first case report of the potentially life-threatening interaction that can occur between docetaxel and the protease inhibitors lopinavir and ritonavir.

**Case presentation:**

A 30-year-old Caucasian woman presented with symptoms suggestive of severe docetaxel toxicity, that is, prolonged myelosuppression, grade 4 mucositis and desquamating rash, following the commencement of post-exposure prophylaxis for a needlestick injury. She had previously received docetaxel chemotherapy with minimal side effects.

**Conclusion:**

This case report highlights a probable and novel drug interaction between docetaxel and lopinavir and/or ritonavir, which is largely unreported in the medical literature. Even though these interactions may be more relevant in the field of HIV medicine, knowledge of these interactions is also beneficial to oncologists and dermatologists, as well as those providing acute medical care.

## Introduction

Docetaxel is a semisynthetic taxane that is widely used in solid tumour oncology including breast, gastric, non-small cell lung and prostate tumour types [1]. Docetaxel is associated with side effects such as fatigue, nausea, vomiting, alopecia, myalgia, skin rashes, oedema, myelosuppression and mucositis [2]. However, docetaxel is usually well tolerated at the dose administered, particularly in patients with no significant comorbidities [2,3]. It acts by inducing microtubular stability by binding tubulin, thus preventing depolymerisation and the normal dynamics of the microtubular network. This results in cell cycle arrest and apoptosis [1,4]. Its pharmacokinetics have been widely studied, and it is well established that it is metabolized to pharmacologically inactive products by the cytochrome P450 3A isoenzymes [5]. Despite this, little has been published regarding the potential interactions of docetaxel with other drugs, and the consequences of such interactions.

## Case presentation

A 30-year-old Caucasian woman with no significant past medical history underwent a wide local excision and sentinel lymph node biopsy for a 15 mm grade 2 invasive ductal carcinoma of the right breast. She was subsequently treated with adjuvant chemotherapy with a modified fluorouracil, epirubicin, cyclophosphamide and docetaxel (Taxotere, Sanofi Aventis) (FEC-T) regimen. The treatment consisted of fluorouracil 600 mg/m^2^, epirubicin 75 mg/m^2^ and cyclophosphamide 600 mg/m^2^ every 21 days for three cycles, followed by sequential three-cycle treatments of 100 mg/m^2^ of docetaxel every 21 days. She received primary prophylaxis with pegylated granulocyte-colony stimulating factor (GCSF) regimen (pegfilgrastim 6 mg subcutaneously 24 hours after chemotherapy). She tolerated the first two cycles of docetaxel well with minimal toxicity (grade 2 fatigue, grade 1-2 nausea and grade 1 neuropathy).

Following the fifth cycle of chemotherapy (second cycle of single agent docetaxel), she sustained a low-risk needlestick injury, with no contact with blood, from her HIV-positive partner. She had previously tested negative in regular HIV tests, most recently 3 months prior to admission, but sought advice from the centre she usually attended. She was commenced on post-exposure prophylaxis (PEP) with Combivir (lamivudine 150 gm and zidovudine 300 mg twice daily, GlaxoSmithKline) and Kaletra (lopinavir 400 mg and ritonavir 100 mg twice daily, Abbott) a week before her third cycle of docetaxel.

She received her third cycle uneventfully with standard steroid prophylaxis (dexamethasone 8 mg twice daily for 3 days commencing the day before treatment). Routine blood tests taken prior to her third cycle revealed a normal full blood count and differential, normal renal function and normal hepatic function. She was admitted on day 6 of the cycle with febrile neutropenia, grade 2 mucositis and grade 2 arthralgia and myalgia. Apart from her recent docetaxel chemotherapy with concomitant steroids and antiretroviral prophylaxis (lamivudine, zidovudine, lopinavir and ritonavir), the patient was taking no other medications. Total white cell count (WCC) was 1.3 × 10^9^/litre with a neutrophil count of 0.6 × 10^9^/litre on admission, which decreased further to 0.005 × 10^9^/litre the following day. Renal and hepatic function tests, including albumin, were within the normal range, and remained within the normal range throughout her admission. She was started on broad spectrum antibiotics with tazocin and gentamycin and antifungal prophylaxis with fluconazole 50 mg daily. Blood cultures sent on admission grew a fully sensitive *Cellumonas* species. Subsequent blood cultures tested negative.

**Figure 1:**
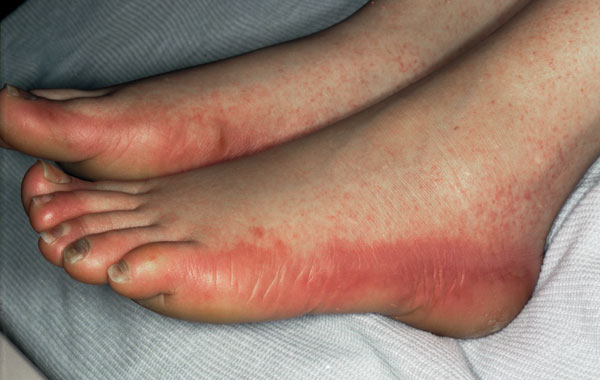
**Appearance of feet (Day 11)**.

**Figure 2:**
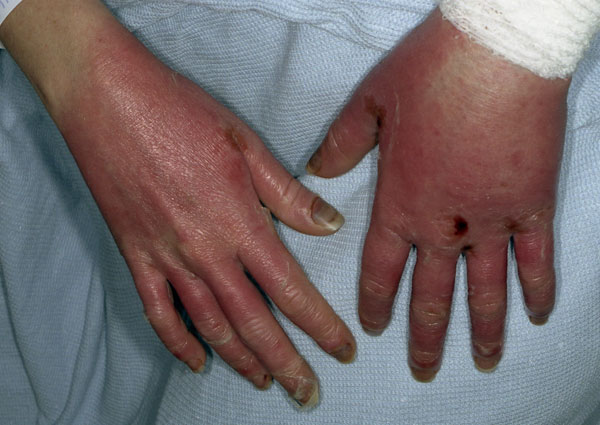
**Desquamating rash on hands (Day 11)**.

Over the next few days, her mucositis worsened to grade 4. She continued to have a swinging pyrexia with persistent grade 4 neutropenia, and was started on teicoplanin, which was then followed by meropenem with continued teicoplanin and gentamicin as per local protocol. The patient received additional daily GCSF and the fluconazole was increased to 200 mg daily. Her neutrophil count recovered to 2.1 × 10^9^/litre on day 7 of her admission. She subsequently developed diarrhea with negative cultures, and on day 10 developed facial swelling and erythema. The combivir was switched to an alternative antiretroviral due to ongoing myelosuppression, but the patient decided to stop taking all antiretrovirals at this point. She then developed a florid rash on her hands and feet (Figures 1 and 2). Due to her ongoing pyrexia and grade 4 mucositis, her fluconazole medication was changed to caspofungin. A high resolution computerised tomography (CT) scan of the patient's thorax tested negative for disseminated fungal infection. Meanwhile, topical treatment was prescribed for her face, hands and feet.

Two weeks after admission, the patient's condition failed to improve. Ganciclovir was added to her medication to address possible cytomegalovirus (CMV) related mucositis. She was also started on total parenteral nutrition. Both CT sinuses and echocardiography performed on the patient yielded negative results, but results of her oesophagogastroduodenoscopy (OGD) and flexible sigmoidoscopy revealed widespread ulceration in the esophagus and gastric antrum that was biopsied. Meanwhile, a galactomannan antigen assay for *Aspergillus* and fungal cultures from her biopsies were negative.

On day 16, the patient's condition began to slowly improve, associated with an improvement in the WCC and resolution of the mucositis. Apart from a mild upper gastrointestinal bleed on day 20, which was managed conservatively, the patient's recovery was sustained. She was finally discharged home well 26 days after admission.

## Discussion

The striking feature of this case was the severity and duration of mucositis, skin reaction, and neutropenia in the presence of normal hepatic function in an individual who had previously tolerated docetaxel-containing chemotherapy. The temporal relationships in this case suggest several aetiological options:

A) A sudden change in the individual's ability to metabolise docetaxel due to intrinsic factors.

B) An adverse reaction either to the retroviral medications or to other supportive medications.

C) A drug interaction between docetaxel and other medications.

Data regarding the pharmaceutical agents discussed below has been obtained from online sources including the British National Formulary (http://www.bnf.org), the National Cancer Institute (http://www.cancer.gov) and Micromedex (www.micromedex.com).

In the absence of altered organ function, as the patient had normal liver and renal function throughout, option A would appear unlikely. The patient was started on several new medications within a short time interval, so option B deserves further consideration. The patient received 13 days of PEP before her admission. The combination of lamivudine and zidovudine is unusually known to cause skin rashes in 9% and neutropenia in 7.2% of patients receiving treatment. The combination of lopinavir and ritonavir can be associated with allergic reactions (<2%), facial oedema (<2%), skin rash (2%), stomatitis and oesophagitis (<2%), and neutropenia in combination with nucleoside reverse transcriptase inhibitors (4%). In the absence of any mitigating factor, it is unusual for a patient with no previous experience of toxicity in any organ system to suddenly experience such severe toxicity.

It is, however, possible that other medications received during the hospital admission, while not the primary cause of the side effects observed, prolonged or exacerbated the patient's toxicity. Examples of this effect would be the association of stomatitis with tazocin and gentamycin, of colitis and diarrhoea with various antibiotics, of neutropenia with teicoplanin and ganciclovir, and of CYP450 3A4 inhibition with fluconazole. It should be noted that the patient's rash had neither the distribution nor characteristic features of Stevens-Johnson syndrome.

Option C is the most likely reason for the toxicity observed because many of the clinical features of the patient's presentation, that is, the onset and duration of neutropenia; development of stomatitis, mucositis and colitis; facial oedema; and characteristics and distribution of the skin rash, resembled severe toxicity to docetaxel. Among patients receiving 100 mg/m^2^ docetaxel with normal liver function and appropriate premedication, febrile neutropenia occurs in up to 11% of cases, severe stomatitis in 7.4% and fluid retention in 64%. Two other factors are implicated in the observed drug reaction: (1) the cytochrome P450 system is both essential for the metabolism of docetaxel into inactive metabolites; and (2) the cytochrome P450 system can be inhibited by the addition of protease inhibitors (PIs).

Antiretroviral therapies, PIs in particular, are known to modulate the cytochrome P450 system. Ritonavir-boosted PI combinations were specifically introduced to exploit the inhibition of cytochrome enzymes by ritonavir, thereby increasing the levels of other PIs such as lopinavir by reducing hepatic metabolism.

As taxanes such as docetaxel are also metabolized by the cytochrome P450 system, the potential for interactions is significant. Pharmacokinetic interactions from altered metabolism via modulation of the cytochrome P450 pathway may result in drug accumulation and increased toxicity or reduced efficacy of one or both drugs. In the absence of pharmacokinetic interactions, the two drugs may still produce clinically relevant pharmacodynamic interactions due to antagonism, additive effects or synergy [6]. A recent study discussed the potential for interactions between antineoplastic agents and highly active antiretroviral therapy (HAART) [5]. Evidence also suggest that patients who receive chemotherapy in conjunction with HAART have improved response rates and survival rates compared with patients who undergo chemotherapy alone [7].

The cytochrome P450 system is essential to the metabolism of docetaxel, and the CYP3A4 route is the sole mechanism of the conversion of docetaxel to several inactive metabolites [8]. It was previously noted that administration of the PI ritonavir in mice increased the plasma levels of oral docetaxel by up to 50 times. The authors thus suggested that the co-administration of ritonavir may be a novel way to increase the bioavailability of oral docetaxel [9].

It is interesting to note that a recent study assessing treatment-related effects via the addition of ritonavir to docetaxel in androgen-independent prostate cancer cell lines revealed that the addition of ritonavir enhanced both the antiproliferative and the proapoptotic effects of docetaxel in DU145 cells [10]. Further analysis of quantitative real-time polymerase chain reaction (PCR) showed that ritonavir completely suppressed docetaxel-induced CYP3A4 expression. The same observation was seen in tumour xenografts in mice. Once again, the addition of ritonavir to chemotherapy was advocated as a potential therapeutic strategy, due to its ability to potentiate levels of docetaxel.

To the best of our knowledge, there is only one other published case report of a patient treated with docetaxel that describes phenomena similar to those we observed in our patient. The report was of a patient undergoing chemotherapy for metastatic breast cancer with low dose docetaxel 36 mg/m^2^ and trastuzumab, concurrently treated with the PI nelfinavir. The patient, who was hospitalized on day three of her first cycle of treatment due to severe neutropenic sepsis and acute respiratory distress syndrome (ARDS), subsequently died [11]. There are also reports of similar phenomena being observed in patients treated with paclitaxel and HAART [11,12], although the consequences may not appear as severe, possibly because the metabolism of paclitaxel is less dependent on the CYP3A4 pathway [5].

More data on the consequences of interactions between HAART and non-taxane containing regimens exist. For example, a recent trial compared the toxicity associated with PI-containing HAART and non-PI HAART in patients receiving infusional cyclophosphamide, doxorubicin and etoposide (CDE) for AIDS-related non-Hodgkin's lymphoma. While no statistically significant difference was found between the two groups in terms of overall survival or response rates, the PI-containing arm experienced more grade 3 and 4 infections (48% versus 25%, χ^2^ test p = 0.0025), more prolonged neutropenia and more delays on chemotherapy [13].

Further support for a potential drug interaction between docetaxel and PIs can be derived from the published literature suggesting interactions with other CYP3A4 modulators. Whilst formal clinical studies in this setting are sparse, *in vivo* and *in vitro* studies suggest that the metabolism of docetaxel can be modified by many drugs that induce or inhibit cytochrome P450 3A4, including erythromycin and ketoconazole [14,15], with the erythromycin breath test advocated as a method of quantifying the degree of CYP3A4 activity following administration of docetaxel [15].

In order to quantify the strength of the association, the authors used the Drug Interaction Probability Scale (DIPS), designed to assess the probability of a causal relationship between a potential drug reaction and an event, and specifically of a drug-drug interaction [16]. Using the data presented above, a score of 5 classified this as a probable causal relationship.

## Conclusion

After other possible aetiologies had been excluded, our final diagnosis was one of severe docetaxel toxicity precipitated by the concomitant administration of PIs as part of PEP. The mechanism of this potential novel drug-drug interaction was concluded to be as a result of the inhibition of CYP3A4 causing an increased and prolonged exposure of normal tissues to docetaxel in its active form.

We initially thought this diagnosis was unlikely as the patient had previously tolerated two cycles at the same dose. However, the patient's main presenting features of prolonged febrile neutropenia with profound myelosuppression, severe mucositis, initial severe myalgia, and severe skin toxicity were entirely consistent with a reaction to docetaxel. We explored a possible causative link between the patient's severe toxicity on this occasion and her concurrent medication with PEP, and found no other predisposing features for docetaxel toxicity, such as abnormal liver function tests [3,17].

This case report highlights the severity of the potential interaction between docetaxel and PI-based antiretroviral therapy, with lopinavir and ritonavir in this instance. While these interactions are perhaps well known to clinicians treating HIV-associated malignancies, they are less obvious when antiretrovirals are prescribed as part of PEP. Clinicians should be aware of this reaction and routinely avoid the combination of PIs with taxanes. Non-PI based regimes may provide a viable alternative, but further studies are required. The relative benefits of PEP need to be considered very carefully in a patient undergoing concomitant taxane-based chemotherapy.

## Abbreviations

ARDS: acute respiratory distress syndrome; CDE: cyclophosphamide doxorubicin and etoposide; CMV: cytomegalovirus; CT: computerised tomography; DIPS: Drug Interaction Probability Scale; FEC-T: fluorouracil, epirubicin, cyclophosphamide and docetaxel (Taxotere); GCSF: granulocyte-colony stimulating factor; HAART: highly active antiretroviral therapy; OGD: oesophagogastroduodenoscopy; PCR: polymerase chain reaction; PEP: post-exposure prophylaxis; PI: protease inhibitor; WCC: white cell count.

## Consent

Written informed consent was obtained from the patient for publication of this case report and any accompanying images. A copy of the written consent is available for review by the Editor-in-Chief of this journal.

## Competing interests

The authors declare that they have no competing interests.

## Authors' contributions

MH, RM, MF and IS were all involved in the management of the patient. MH prepared the manuscript. IS revised and edited the manuscript. All authors read and approved the final manuscript.
